# Development and validation of a new staging system for node‐negative gastric cancer based on recursive partitioning analysis: An international multi‐institutional study

**DOI:** 10.1002/cam4.2170

**Published:** 2019-05-08

**Authors:** Jian‐Xian Lin, Zu‐Kai Wang, Wei Wang, Jian‐Wei Xie, Jia‐Bin Wang, Jun Lu, Qi‐Yue Chen, Long‐Long Cao, Mi Lin, Ru‐Hong Tu, Chao‐Hui Zheng, Ping Li, Zhi‐Wei Zhou, Chang‐Ming Huang

**Affiliations:** ^1^ Department of Gastric Surgery Fujian Medical University Union Hospital Fuzhou China; ^2^ Key Laboratory of Ministry of Education of Gastrointestinal Cancer Fujian Medical University Fuzhou China; ^3^ Department of Gastric and Pancreatic Surgery Sun Yat‐sen University Cancer Center Guangzhou China

**Keywords:** gastric cancer, node‐negative, recursive partitioning analysis, TNM staging system

## Abstract

**Background:**

Whether the tumor‐node‐metastasis (TNM) staging system is appropriate for patients with node‐negative gastric cancer (GC) is still inconclusive. The modified staging system developed by recursive partitioning analysis (RPA) showed good prognostic performance in a variety of cancers. The application of RPA has not been reported in the prognostic prediction of GC.

**Methods:**

Node‐negative GC patients who underwent radical resection at Fujian Medical University Union Hospital (n = 862) and Sun Yat‐sen University Cancer Center (n = 311) with at least 5 years of follow‐up were selected as the training set. RPA was used to develop a modified staging system. Patients from the Surveillance, Epidemiology, and End Results database (n = 1415) were selected as the validation set.

**Results:**

The 5‐year overall survival (OS) rates of patients with 8th AJCC‐TNM stage IA‐IIIA in the training set were IA 95.2%, IB 87.1%, IIA 78.3%, IIB 75.8%, and IIIA 72.6%. Multivariate analysis (MVA) showed that larger tumor size, elder age, and deeper depth of invasion were independent predictors for OS in patients with node‐negative GC (all *P* < 0.05). Patients were reclassified into RPA I, RPA II, RPA III, and RPA IV stages based on RPA; the 5‐year OS rates were 96.1%, 87.2%, 81.0%, and 64.3%, respectively, with significant difference (*P* < 0.05). Two‐step MVA showed that the RPA staging system was an independent predictor of OS (*P* < 0.05). Compared with the 8th AJCC‐TNM staging system, the RPA staging system had a smaller AIC value (2544.9 vs 2576.2), higher χ^2^ score (104.2 vs 69.6) and higher Harrell's C‐index (0.697 vs 0.669, *P* = 0.007). The similar results were found in the validation set.

**Conclusions:**

A new prognostic predictive system based on RPA was successfully developed and validated, which may be suggested for staging node‐negative GC in future.

## 1 INTRODUCTION

Gastric cancer (GC) is one of the most common malignant tumors of the digestive system, is the 5th most common malignant tumor and is the 3rd leading cause of cancer‐related mortality.^1^ The current postoperative prognosis assessment and subsequent treatment decisions for GC are based on the tumor‐node‐metastasis (TNM) staging system proposed by the American Joint Committee on Cancer (AJCC). In the past decades, the TNM staging system has been revised several times in order to predict the prognosis more accurately. The 8th edition of the AJCC staging system was released in October 2016 and was recommended as a replacement for the older version in 2018.^2^ Some studies on the prognostic predictive performance of this latest version show that the 8th AJCC‐TNM stage system can better predict the prognosis of patients with GC.^3^, ^4^, ^5^, ^6^, ^7^

With the same tumor stage (T stage), node‐negative GC patients have a higher survival rate than node‐positive patients. However, in the TNM staging system, the staging factor of node‐negative GC patients has only T stage. Is it reasonable to incorporate patients into the TNM staging system only based on T stage? Deng et al found that the 5‐year survival rates of node‐negative GC patients with 7th AJCC‐TNM stage IB and IIA were similar (70.4% vs 71.9%).^8^

Recursive partitioning analysis (RPA) was first proposed by Goldman et al in 1982 and has been widely used in medical decision‐making. After continuous development, RPA changed from the earliest classification tool to a simple and intuitive model for prognosis.^9^, ^10^, ^11^, ^12^ At present, the model has been successfully applied to head and neck cancer, thyroid cancer, breast cancer and prostate cancer to develop modified staging systems.^11^, ^12^, ^13^, ^14^, ^15^, ^16^, ^17^ In the prognostic assessment of GC, the application of RPA has not been reported.

Therefore, based on the long‐term follow‐up information of large sample data, this study tried to develop a new staging system for node‐negative GC using RPA.

## 2 MATERIAL AND METHODS

### 2.1 Patients

This study retrospectively analyzed the clinicopathological data of patients from Fujian Medical University Union Hospital (FMUUH) and Sun Yat‐sen University Cancer Center (SYSUCC) who underwent radical gastrectomy (FMUUH, January 1994 to June 2012; SYSUCC, January 1990 to December 2012). The inclusion criteria were as follows: (a) number of examined nodes >15; (b) no evidence of distant metastasis; and (c) no lymph node metastasis. Patients were excluded if they (a) had received neoadjuvant therapy; (b) had multiple primary cancers; (c) died within 3 months after operation; or (d) had incomplete clinical or pathological information. Finally, 1173 patients were included in this study as the training set (FMUUH, n = 862; SYSUCC, n = 311) (Supplementary Figure S1). The type of surgical resection and the extent of lymph node dissection were selected according to the Japanese Gastric Cancer Association.^18^ Six cycles of fluoride‐based adjuvant chemotherapy were recommended for all patients with stage II or III GC. The T stage, N stage and final stage of all study patients were classified according to the 8th edition of the AJCC‐TNM staging system.^2^ Follow‐up evaluation after surgery generally consisted of clinic visits, with labs and computed tomography (CT) scans repeated every 3‐6 months for the first 2 years and every 6‐12 months for the following 3‐5 years, then annually afterward. The survival time was recorded from the date of surgery to the last follow‐up date, date of death, or date until the end of follow‐up in the database (such as loss to follow‐up or death due to other diseases). The median follow‐up time of the training set was 81.0 months. The study was approved by the FMUUH and SYSUCC Institutional Review Board.

### 2.2 Recursive partitioning analysis

RPA is a statistical method for multivariate analysis (MVA) that separates patients into different homogeneous risk groups to determine predictors for survival.^19^ RPA in this study was carried out using the R package "rpart". The algorithm selects the predictor that provides the best or “optimal” split, such that each of the two subgroups is more homogeneous with respect to outcome. Each subgroup is further dichotomized into smaller and more homogeneous groups by choosing the variable that best splits the subgroup. Iterative splits continued until too few values for additional splits. The pruning procedure was then used on the original partitioning tree in an attempt to cut the tree back to the point where the overall predictive accuracy was maximized, thereby preventing data over fitting.^20^ The analysis was performed with minimal bucket size of 90, minimum split size of 200 and complexity parameter of 0.01. The 5‐year overall survival (OS) rate of each RPA stage was calculated, and the prognostic performance of the RPA staging system was compared with the 8th AJCC‐TNM staging system.

### 2.3 External validation population

An external validation dataset was obtained from the Surveillance, Epidemiology, and End Results (SEER) from January 1988 to December 2008 (Supplementary Fig. S2). Inclusion criteria included the following: (a) pathologically confirmed gastric adenocarcinoma; (b) underwent radical gastrectomy; (c) no evidence of distant metastases; and (d) no lymph node metastasis. Exclusion criteria were as follows: (a) number of examined nodes ≤15; (b) death within 3 months after operation; and (c) incomplete clinical pathological information. Finally, 1415 patients were included in this study as an external validation set. The median follow‐up time of the SEER validation set was 109.0 months.

### 2.4 Statistical analysis

Categorical variables were analyzed using the Chi‐square test or Fisher's exact test, whereas continuous variables were analyzed using Student's *t* test or Mann‐Whitney *U* test. Univariate and multivariate analyses were performed using Cox regression analysis; survival estimates were reported as hazard ratios (HRs) with 95% confidence intervals (95% CIs). Based on the results of the MVA, RPA was used to divide the patients into different risk groups. The Kaplan‐Meier method was used to estimate the time‐dependent OS probabilities. The log‐rank test was used for statistical comparisons of the survival curves. A two‐step MVA was performed to investigate the validity of the RPA staging system.^21^ In the 1st step of the MVA, all the significant factors in the univariate analysis were included as well as the 8th AJCC‐TNM staging system, excluding the RPA staging system. In the 2nd step of the MVA, both the 8th AJCC‐TNM and the RPA staging system were included. The relative discriminatory abilities of the different staging systems were assessed using the likelihood ratio Chi‐square test, the Akaike Information Criteria (AIC) and the Harrell's concordance index (C‐index). A higher likelihood ratio Chi‐square (*χ* ^2^) score means better homogeneity; smaller AIC values represent better optimistic prognostic stratification. A higher C‐index indicates a better discriminatory ability. Statistical analysis was performed using SPSS 22.0 software (SPSS, Chicago, IL, USA) and R 3.4.0 software (The R Foundation for Statistical Computing, Vienna, Austria). All statistical tests were two‐sided, and a *P* value <0.05 was considered statistically significant.

## 3 RESULTS

### 3.1 Patient characteristics

The training set included a total of 1173 patients from FMUUH and SYSUCC. The baseline characteristics are shown in Table 1. There were 857 (73.1%) males and 316 (26.9%) females. The mean age was 58.1 ± 11.7 years, and the mean tumor size was 36.2 ± 21.9 mm. The majority of patients (38.2%) had tumors located in the antrum/pylorus, 867 (73.9%) had an undifferentiated histological type, and more than half (68.7%) had no lymphovascular invasion. Nearly half of patients had the tumor confined to the mucosa or submucosa (8th AJCC‐TNM stage IA), the mean number of examined nodes was 28 ± 9.4, and at least 29.1% of patients received postoperative adjuvant chemotherapy.

**Table 1:** Characteristics of the training and validation data set

Clinicopathological Features	Training Set (n = 1173)		Validation Set (n = 1415)	
	Mean or n	SD or %	Mean or n	SD or %
Gender				
Male	857	73.1	791	55.9
Female	316	26.9	624	44.1
Age, mean (SD), year	58.1	11.7	66.5	13
Tumor size, mean (SD), mm	36.2	21.9	43.2	30
Tumor site				
Cardia/fundus	335	28.6	71	5
Body	330	28.1	202	14.3
Antrum/pylorus	448	38.2	501	35.4
Overlapping regions	60	5.1	406	28.7
Lesser/greater curvature	/	/	109	7.7
Stomach NOS	/	/	126	8.9
Histological type				
Differentiated	306	26.1	523	37
Undifferentiated	867	73.9	892	63
Lymphovascular invasion				
Absent	806	68.7	Missing	/
Present	56	4.8		/
Unknown	311	26.5		/
Depth of invasion				
T1	501	42.7	537	38
T2	211	18	261	18.4
T3	136	11.6	448	31.7
T4	325	27.7	169	11.9
8th AJCC‐TNM stage				
IA	501	42.7	537	38
IB	211	18	261	18.4
IIA	136	11.6	448	31.7
IIB	283	24.1	111	7.8
IIIA	42	3.6	58	4.1
No. examined nodes, mean (SD)	28	9.4	25.1	10.1
Adjuvant chemotherapy				
No/unknown^a^	832	70.9	1172	82.8
Yes	341	29.1	243	17.2

Abbreviations: SD, standard deviation; NOS, not otherwise specified; AJCC, American Joint Committee on Cancer; TNM stage: tumor‐node‐metastasis stage.

a Including patients without adjuvant chemotherapy and unknown status of adjuvant chemotherapy. Because the information for adjuvant chemotherapy was not available in the SYSUCC database and was only recorded as “No/Unknown” or “Yes” in the SEER database.

In addition, 1415 patients from SEER were included in the study as an external validation set. In the SEER cohort, there were 791 (55.9%) males and 624 (44.1%) females. The mean age was 66.5 ± 13.0 years, and the mean tumor size was 43.2 ± 30.0 mm. The tumor site and TNM stage were similar to those of the training set. The mean number of examined nodes was 25.1 ± 10.1, and at least 17.2% of patients received postoperative adjuvant chemotherapy. Detailed baseline characteristics are shown in Table 1.

### 3.2 Univariate and multivariate Cox regression analysis for overall survival

**Figure 1:**
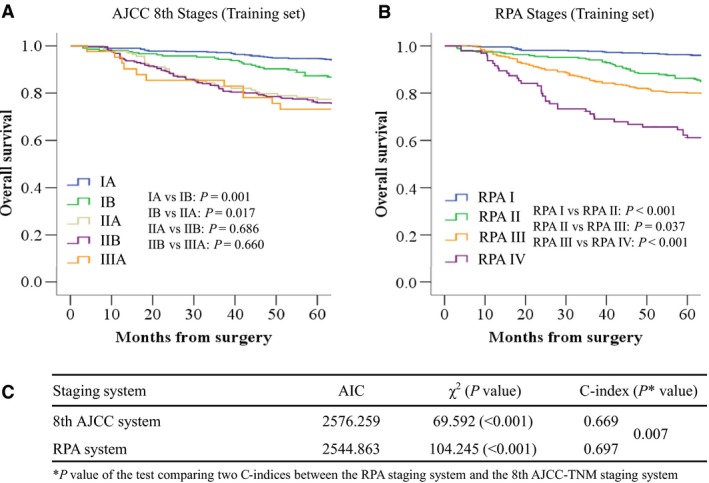
A, Kaplan‐Meier estimates of overall survival stratified by 8th AJCC‐TNM staging system. B, Kaplan‐Meier estimates of overall survival stratified by RPA staging system. C, Performances of different staging systems for gastric cancer in the training set

In the 8th AJCC‐TNM staging system, the 5‐year OS rates for each stage were as follows: IA 95.2%, IB 87.1%, IIA 78.3%, IIB 75.8% and IIIA 72.6% (IIA vs IIB: *P* = 0.686; IIB vs IIIA: *P* = 0.660; *P* < 0.05 between the other stages) (Figure 1A). Univariate analysis showed that postoperative OS was closely associated with age, tumor size, tumor site, and depth of invasion (all *P* < 0.05). There was no significant correlation between gender, histological type, lymphovascular invasion, number of examined nodes, and postoperative OS. Further MVA showed that age, tumor size, and depth of invasion were independent predictors of postoperative OS (all *P* < 0.05). Larger tumor size, older age, and deeper depth of invasion indicated poor prognosis (Table 2).

**Table 2:** Univariate and multivariate cox regression analysis for overall survival

Parameters	Univariate analysis		Multivariate analysis	
	Hazard Ratio (95% CI)	*P*	Hazard ratio (95% CI)	*P*
Gender				
Male	Ref			
Female	0.776 (0.554‐1.086)	0.139		
Age				
Continuous	1.026 (1.013‐1.040)	<0.001	1.020 (1.007‐1.034)	0.002
Tumor size				
Continuous	1.021 (1.016‐1.026)	<0.001	1.012 (1.006‐1.018)	<0.001
Tumor site		<0.001		0.631
Cardia/fundus	Ref		Ref	
Body	0.685 (0.482‐0.972)	0.034	0.935 (0.654‐1.337)	0.714
Antrum/pylorus	0.488 (0.341‐0.697)	<0.001	0.784 (0.542‐1.135)	0.198
Overlapping regions	0.975 (0.541‐1.758)	0.933	0.959 (0.528‐1.742)	0.890
Histological type				
Differentiated	Ref			
Undifferentiated	1.200 (0.541‐1.758)	0.292		
Lymphovascular invasion		0.449		
Absent	Ref			
Present	1.031 (0.525‐2.025)	0.929		
Unknown	0.809 (0.579‐1.130)	0.214		
pT stage		<0.001		<0.001
T1	Ref		Ref	
T2	2.257 (1.403‐3.631)	0.001	1.873 (1.155‐3.039)	0.011
T3	3.791 (2.362‐6.083)	<0.001	2.436 (1.464‐4.050)	0.001
T4	4.416 (2.990‐6.522)	<0.001	2.971 (1.920‐4.597)	<0.001
No. of examined nodes				
Continuous	1.004 (0.990‐1.020)	0.562		

Abbreviations: CI, confidence interval; Ref, reference; pT stage, pathological tumor stage

### 3.3 RPA staging system

**Figure 2:**
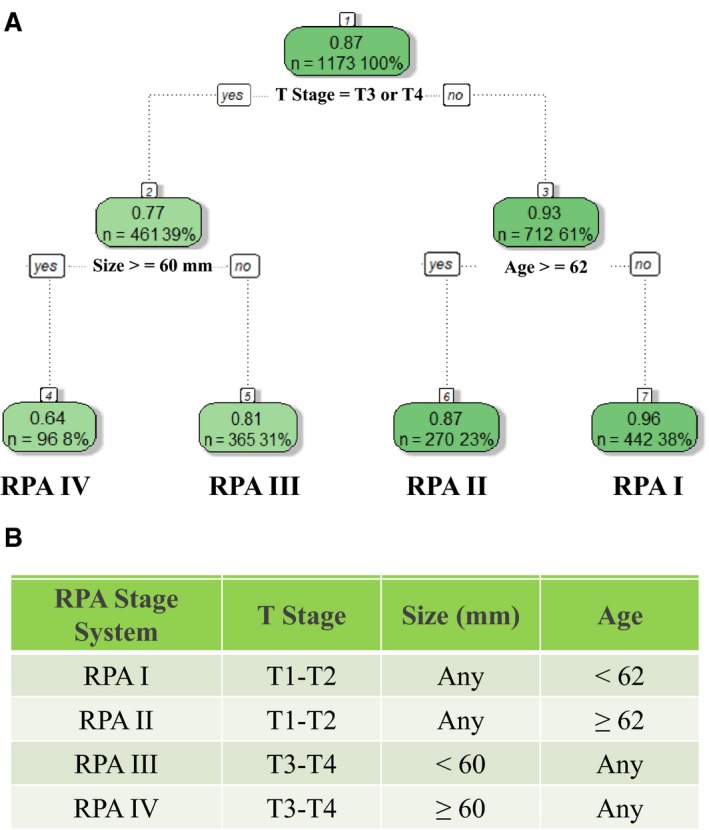
Proposed RPA staging system. A, Proposed stage grouping derived by recursive partitioning analysis (RPA). RPA I: T1‐T2, age <62, regardless of tumor size; RPA II: T1‐T2, age ≥62, regardless of tumor size; RPA III: T3‐T4, tumor size <60 mm, regardless of age; RPA IV: T3‐T4, tumor size ≥60 mm, regardless of age. B, Grid for proposed stage

Based on the results of the MVA, RPA was performed to reclassify the patients in the training set into different groups in accordance with similar 5‐year OS rates. The three independent factors included in the RPA were age, tumor size and depth of invasion. According to the R software prioritization of independent variables, patients in the training set were ultimately divided into 4 groups (RPA I‐IV stage). There were 442 (38%) RPA I stage patients (T1‐T2, age < 62, regardless of tumor size), 270 (23%) RPA II stage patients (T1‐T2, age ≥62, regardless of tumor size), 365 (31%) RPA III stage patients (T3‐T4, tumor size <60 mm, regardless of age) and 96 (8%) RPA IV stage patients (T3‐T4, tumor size ≥60 mm, regardless of age). The 5‐year OS rates for RPA I‐IV stages were 96.1%, 87.2%, 81.0%, and 64.3%, respectively (Figure 2). Sixty‐two point five percent of patients with stage IA and 61.1% of patients with stage IB were reclassified as stage RPA I; 37.5% of patients with stage IA and 38.9% of patients with stage IB were reclassified as stage RPA II; 80.9% of patients with stage IIA, 82.3% of patients with stage IIB and 52.4% of patients with stage IIIA were reclassified as stage RPA III; 19.1% of patients with stage IIA, 17.7% of stage IIB and 47.6% of stage IIIA were reclassified as stage RPA IV (Supplementary Table S1).

### 3.4 Comparison of prognostic performance between the RPA staging system and the 8th AJCC‐TNM staging system

Two‐step MVA was used to identify the validity of the RPA staging system. In the 1st step of the MVA, age, tumor size and the 8th AJCC‐TNM staging system were confirmed to be independent prognostic factors (all *P* < 0.05). The 2nd step of the MVA included the RPA staging system and showed that age and RPA staging system were independent predictors of OS (all *P* < 0.05), while the 8th AJCC‐TNM staging system was no longer significant (Table 3).

**Table 3:** Univariate and two‐step multivariate analyses of the prognostic factors for gastric cancer patients without lymph node metastasis

Parameters	Univariate analysis		Multivariate analysis 1		Multivariate analysis 2	
	HR (95% CI)	*P*	HR (95% CI)	*P*	HR (95% CI)	*P*
Gender (male vs female)	0.776 (0.554‐1.086)	0.139				
Age^a^	1.026 (1.013‐1.040)	<0.001	1.021 (1.008‐1.034)	0.001	1.014 (1.001‐1.028)	0.033
Tumor size^a^	1.021 (1.016‐1.026)	<0.001	1.013 (1.006‐1.019)	<0.001	1.007 (1.000‐1.014)	0.065
Tumor site (U vs M vs L vs Overlapping)	0.785 (0.669‐0.920)	0.003	0.915 (0.785‐1.066)	0.255	0.923 (0.792‐1.076)	0.306
Histological type (differentiated vs undifferentiated)	1.200 (0.541‐1.758)	0.292				
Lymphovascular invasion (absent vs present vs unknown)	0.903 (0.766‐1.065)	0.227				
No. of examined nodes^a^	1.004 (0.990‐1.020)	0.562				
8th AJCC‐TNM stage (IA vs IB vs IIA vs IIB vs IIIA)	1.522 (1.371‐1.690)	<0.001	1.338 (1.183‐1.513)	<0.001	1.060 (0.878‐1.278)	0.546
RPA stage (RPA I vs RPA II vs RPA III vs RPA IV)	2.028 (1.750‐2.351)	<0.001	N/A	N/A	1.623 (1.204‐2.187)	0.001

Note

For multivariate model 1, all the significant factors in the univariate analysis as well as the 8th AJCC‐TNM staging system were included; the RPA staging system was excluded. For multivariate model 2, the RPA staging system was also included

Abbreviations: HR, hazard ratio; CI, confidence interval; U: upper region (cardia/fundus); M: middle region (body); L: lower region (antrum/pylorus); AJCC: American Joint Committee on Cancer; TNM stage: tumor‐node‐metastasis stage; RPA: recursive partitioning analysis

a Continuous variable

Further comparison of the two staging systems showed that the RPA staging system had a smaller AIC value (2544.9 vs 2576.2), higher *χ* ^2^ score (104.2 vs 69.6) and higher Harrell's C‐index (0.697 vs 0.669, *P* = 0.007) (Figure 1C). The statistical assessment of the predictive performance of the two staging systems revealed that the RPA staging system was superior to the 8th AJCC‐TNM staging system.

### 3.5 External validation

**Figure 3:**
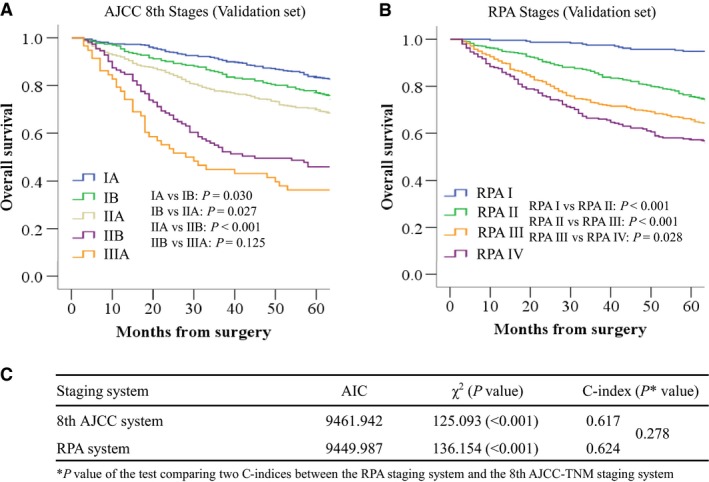
A, Kaplan‐Meier estimates of overall survival stratified by 8th AJCC‐TNM staging system. B, Kaplan‐Meier estimates of overall survival stratified by RPA staging system. C, Performances of different staging systems for gastric cancer in the validation set

The RPA staging system was then verified using the SEER external validation set. In the 8th AJCC‐TNM staging system, the 5‐year OS rates for each stage were as follows: IA 84.2%, IB 78.1%, IIA 69.9%, IIB 46.4% and IIIA 35.8% (IIB vs IIIA: *P* = 0.125, *P* < 0.05 between the other stages) (Figure 3A). The 5‐year OS rates for each stage of RPA staging system were as follows: RPA I 95%, RPA II 76%, RPA III 66%, and RPA IV 57% (all *P* < 0.001) (Figure 3B). Survival differences between each pair of stages were more obviously discriminated in the RPA staging system than the 8th AJCC‐TNM staging system (Figure 3B). The RPA staging system had a smaller AIC value (9450.0 vs 9461.9), higher *χ* ^2^ score (136.2 vs 125.1) and higher Harrell's C‐index (0.624 vs 0.617, *P* = 0.278) (Figure 3C). The superiority of the RPA staging system to the 8th AJCC‐TNM staging system was validated in the SEER external validation set.

## 4 DISCUSSION

To predict the prognosis more accurately, as well as to guide the treatment of malignant tumors, AJCC and the Union for International Cancer Control have established the internationally accepted TNM staging system. From the earliest first edition in 1968 to the present, it has become the standard for clinicians and medical workers to stage malignant tumors. Since the 8th AJCC‐TNM staging system for GC has been recommended to replace the older version in 2018, some scholars have studied its prognostic performance. Ji et al analyzed the survival data of 1663 patients after radical gastrectomy and compared the different prognostic performances of the 7th and 8th AJCC‐TNM staging system. It was found that, regardless of homogeneity, discriminatory ability and monotonicity of gradients, the 8th edition is superior to the 7th edition.^5^ Lu et al analyzed 10 194 Western and 2355 Eastern patients' data and found that for noncardiac cancer patients with a number of examined nodes ≥ 15, the 8th AJCC‐TNM staging system showed better prognostic performance than the 7th.^7^ However, in patients with node‐negative GC, it is not clear whether the TNM staging system is still applicable because it is impossible to assess the impact of N stage on these patients. Li et al found that node‐negative GC patients with a number of examined nodes ≤15 had a similar prognosis as N1 patients, so they hypothesized a new N stage incorporating these patients into the N1 stage. The hypothetical N stage had higher linear trend and likelihood ratio *χ* ^2^scores and smaller AIC values compared with those for the AJCC N stage.^22^ Deng et al found that the number of examined nodes has a stronger predictive value for prognosis than depth of invasion, tumor size, and type of gastrectomy for node‐negative GC.^8^ However, the abovementioned studies did not explicitly develop an appropriate staging system for patients with node‐negative GC, and all of them included cases with a number of examined nodes ≤15. Because of the stage migration, these results may not be as accurate. Therefore, this study included cases with a number of examined nodes >15 in order to develop a new staging system for patients with node‐negative GC using RPA based on large sample data with long‐term follow‐up and to explore whether it is of more accuracy in predicting prognosis than TNM staging system. Recently, Dimitriou et al used pathological automated image analysis technology to extract pathological features of the tumor microenvironment in postoperative specimens, and combined with machine learning methods, described a system that can predict the survival outcome of patients with stage II colorectal cancer accurately.^23^ The accuracy of 5‐year survival rate and 10‐year survival rate predicted by this system were significantly higher than by the pT stage. The purpose of our study is to create a more accurate prognostic prediction tool for patients with malignant tumors, which is similar with Dimitriou et al's.

As a multivariate statistical method, RPA can divide each variable included in the model based on the best or “optimal” split. Because RPA can intuitively generate a concise decision tree with higher sensitivity or/and specificity, it is widely used in medical decision making. RPA was first proposed by Goldman et al in 1982; after many scholars' modifications, the current model is widely used in the prognostic analysis of a variety of malignant tumors, especially head and neck cancer. Huang et al found that for HPV‐related oropharyngeal cancer, the 7th AJCC‐TNM staging system cannot distinguish the prognosis gap between different stages. In contrast, the modified staging system established by RPA showed good prognostic performance.^16^ Dahlstrom et al combined the T stage of oropharyngeal cancer with the N stage of nasopharyngeal cancer and established a modified TNM stage system based on RPA for HPV‐related oropharyngeal cancer, which showed better prognostic performance than the traditional TNM stage system.^14^ The results of our study showed that, in the training set, the 8th TNM staging system cannot distinguish the prognosis difference of each pair of stages for patients with node‐negative GC (IIA vs IIB: *P* = 0.686, IIB vs IIIA: *P* = 0.660, *P* < 0.05); therefore, RPA was used to develop an RPA staging system for N0 patients. Two‐step MVA showed that RPA staging was an independent predictor of OS for patients with node‐negative GC in the training set. In addition, compared with the 8th TNM staging system, the RPA staging system had a smaller AIC value, higher *χ* ^2^ score and Harrell's C‐index. Similar results were obtained when using Western patients' data as an external validation set. Although there was no significant difference in C‐indices between the two staging systems in the validation set (*P* = 0.278), the survival differences between each pair of stages were more obviously discriminated in the RPA staging system than the 8th AJCC‐TNM staging system (all *P* < 0.05). These statistical assessments revealed that the RPA staging system was superior to the 8th AJCC‐TNM staging system for N0 patients.

In previous studies, other approaches such as nomogram and the Cancer Data Clustering Integration Algorithm (EACCD) were also applied to identify independent prognostic factors. Nomogram is a graphical prediction tool based on the Cox proportional hazard model that attempts to combine all proven prognostic factors and quantify the risk as accurately as possible.^24^ Because it does not require risk factors to be independent of each other, RPA outperforms the proportional risk model in identifying prognostic factors, and as a nonparametric technique, it makes no requirement on the underlying distribution of variables. Hence, it relies on fewer modeling assumptions. In addition, because RPA is designed to divide subjects based on the length of survival, it defines different risk groups, while Cox regression models do not. Recently, Hueman et al described a new machine learning based approach to develop a prognostic system for breast cancer.^25^ The method used the EACCD to cluster patients according to their survival and output a dendrogram showing details on the change of survival rates as factor levels vary. The patients were further dividing into different prognostic groups according to the C‐index by cutting the dendrogram. This approach is similar to the RPA of this study: patients were both stratified according to prognostic factors, and different risk groups were generated based on the length of survival. However, there are still several differences as followed. First, in EACCD, age was included for analysis as categorical variables, while in this study we included age and tumor size in the form of continuous variables into the RPA to obtain the best prognostic cut‐off point; Second, in Hueman et al's study, the best prognostic groups were determined by calculating the C‐index, but in our study, we set appropriate parameters such as minimum bucket size, minimum split size and complexity parameter to achieve the purpose of “pruning”, thus preventing the model from over fitting; Third. EACCD is an unsupervised learning method for data analysis without training set and validation set, while RPA is a supervised learning method, which should develop prognostic model in training set and then verify the reliability of the model in the validation set.

The large sample size and the long follow‐up duration of this study lend reliability to the results. Nevertheless, there are several limitations to this study. First, limited by its retrospective nature, prospective studies with large sample sizes are still needed to confirm our results. Second, although the SEER database maintains highly accurate records and is usually used for external validation, incorrect and missing data are still possible. Third, postoperative adjuvant chemotherapy may affect the prognosis of GC patients. The exact information for adjuvant chemotherapy was not available in the SYSUCC database and the SEER database which may have a certain impact on the prognostic performance of the RPA staging systems. Nevertheless, we applied RPA for the first time in the prognostic analysis of patients with node‐negative GC and developed an appropriate staging system with better predictive performance than the 8th AJCC‐TNM staging system. Furthermore, the utility and validity of the RPA staging systems were verified in a Western external validation set.

## CONFLICT OF INTEREST

There are no conflicts of interest or financial ties to disclose from any of author.
