# Supplementary material for: Development and validation of a new staging system for node‐negative gastric cancer based on recursive partitioning analysis: An international multi‐institutional study
Source: Cancer Med. 2019 May 8;8(6):2962–70. doi: 10.1002/cam4.2170 (PMC6558615; doi:10.1002/cam4.2170)
Supplement: Supplementary file 1 [file CAM4-8-2962-s001.pptx]

## Slide 1
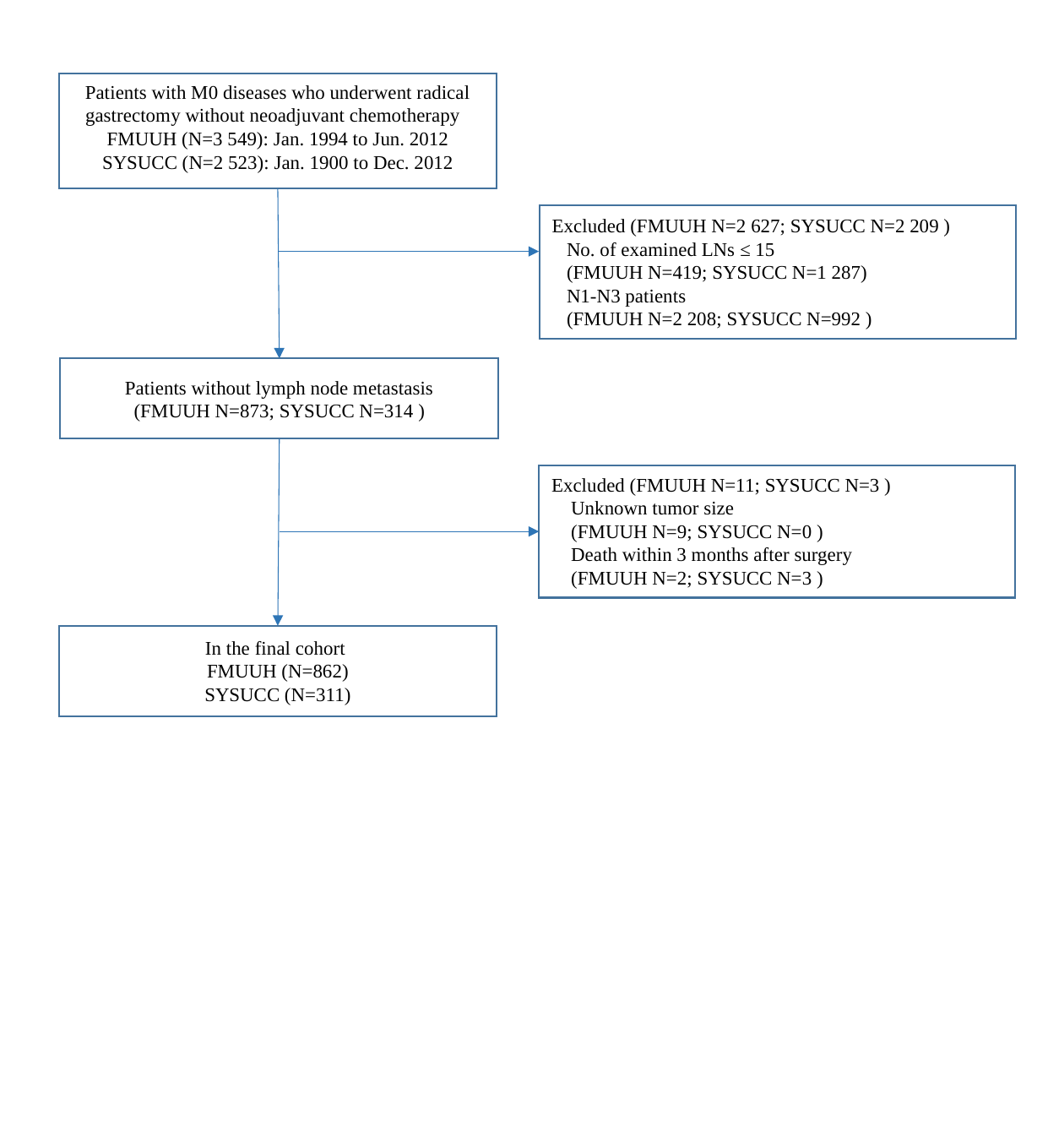

Patients with M0 diseases who underwent radical gastrectomy without neoadjuvant chemotherapy
FMUUH (N=3 549): Jan. 1994 to Jun. 2012
SYSUCC (N=2 523): Jan. 1900 to Dec. 2012
Excluded (FMUUH N=2 627; SYSUCC N=2 209 )
 No. of examined LNs ≤ 15
 (FMUUH N=419; SYSUCC N=1 287)
 N1-N3 patients
 (FMUUH N=2 208; SYSUCC N=992 )
Patients without lymph node metastasis
(FMUUH N=873; SYSUCC N=314 )
Excluded (FMUUH N=11; SYSUCC N=3 )
 Unknown tumor size
 (FMUUH N=9; SYSUCC N=0 )
 Death within 3 months after surgery
 (FMUUH N=2; SYSUCC N=3 )
In the final cohort
FMUUH (N=862)
SYSUCC (N=311)
