# Supplementary material for: Development and validation of a new staging system for node‐negative gastric cancer based on recursive partitioning analysis: An international multi‐institutional study
Source: Cancer Med. 2019 May 8;8(6):2962–70. doi: 10.1002/cam4.2170 (PMC6558615; doi:10.1002/cam4.2170)
Supplement: Supplementary file 2 [file CAM4-8-2962-s002.pptx]

## Slide 1
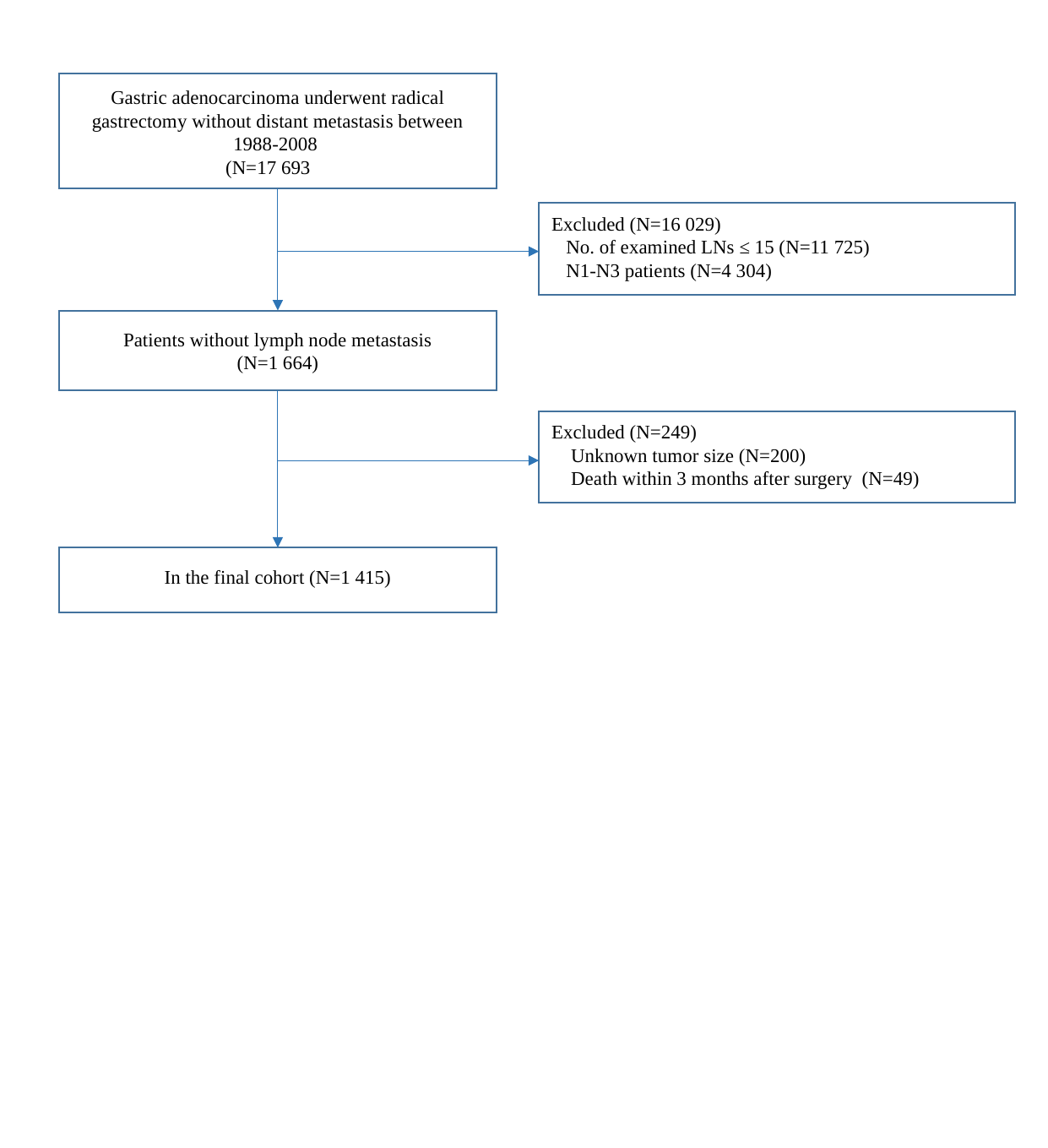

Gastric adenocarcinoma underwent radical gastrectomy without distant metastasis between 1988-2008
(N=17 693）
Excluded (N=16 029)
 No. of examined LNs ≤ 15 (N=11 725)
 N1-N3 patients (N=4 304)
Patients without lymph node metastasis
(N=1 664)
Excluded (N=249)
 Unknown tumor size (N=200)
 Death within 3 months after surgery (N=49)
In the final cohort (N=1 415)
