# Supplementary material for: Development and validation of a new staging system for node‐negative gastric cancer based on recursive partitioning analysis: An international multi‐institutional study
Source: Cancer Med. 2019 May 8;8(6):2962–70. doi: 10.1002/cam4.2170 (PMC6558615; doi:10.1002/cam4.2170)
Supplement: Supplementary file 3 [file CAM4-8-2962-s003.docx]

Supplementary Table 1. Distribution of 8^th^ AJCC-TNM stages within each RPA stage in the training set.

|  | IA | IB | IIA | IIB | IIIA | Total |
| --- | --- | --- | --- | --- | --- | --- |
|  | (n *=* 501) | (n = 211) | (n = 136) | (n = 283) | (n = 42) | (n *=* 1173) |
| RPA I | 313 (62.5) | 129 (61.1) | 0 (0) | 0 (0) | 0 (0) | 442 (37.7) |
| RPA II | 188 (37.5) | 82 (38.9) | 0 (0) | 0 (0) | 0 (0) | 270 (23.0) |
| RPA III | 0 (0) | 0 (0) | 110 (80.9) | 233 (82.3) | 22 (52.4) | 365 (31.1) |
| RPA IV | 0 (0) | 0 (0) | 26 (19.1) | 50 (17.7) | 20 (47.6) | 96 (8.2) |

Values in parentheses are percentages
